# Fusion of fruit image processing and deep learning: a study on identification of citrus ripeness based on R-LBP algorithm and YOLO-CIT model

**DOI:** 10.3389/fpls.2024.1397816

**Published:** 2024-06-05

**Authors:** Chenglin Wang, Qiyu Han, Chunjiang Li, Tianlong Zou, Xiangjun Zou

**Affiliations:** ^1^ Faculty of Modern Agricultural Engineering, Kunming University of Science and Technology, Kunming, China; ^2^ Foshan-Zhongke Innovation Research Institute of Intelligent Agriculture and Robotics, Guangzhou, China; ^3^ College of Intelligent Manufacturing and Modern Industry, Xinjiang University, Wulumuqi, China

**Keywords:** citrus, ripeness identification, deep learning, image processing, LBP feature

## Abstract

Citrus fruits are extensively cultivated fruits with high nutritional value. The identification of distinct ripeness stages in citrus fruits plays a crucial role in guiding the planning of harvesting paths for citrus-picking robots and facilitating yield estimations in orchards. However, challenges arise in the identification of citrus fruit ripeness due to the similarity in color between green unripe citrus fruits and tree leaves, leading to an omission in identification. Additionally, the resemblance between partially ripe, orange-green interspersed fruits and fully ripe fruits poses a risk of misidentification, further complicating the identification of citrus fruit ripeness. This study proposed the YOLO-CIT (You Only Look Once-Citrus) model and integrated an innovative R-LBP (Roughness-Local Binary Pattern) method to accurately identify citrus fruits at distinct ripeness stages. The R-LBP algorithm, an extension of the LBP algorithm, enhances the texture features of citrus fruits at distinct ripeness stages by calculating the coefficient of variation in grayscale values of pixels within a certain range in different directions around the target pixel. The C3 model embedded by the CBAM (Convolutional Block Attention Module) replaced the original backbone network of the YOLOv5s model to form the backbone of the YOLO-CIT model. Instead of traditional convolution, Ghostconv is utilized by the neck network of the YOLO-CIT model. The fruit segment of citrus in the original citrus images processed by the R-LBP algorithm is combined with the background segment of the citrus images after grayscale processing to construct synthetic images, which are subsequently added to the training dataset. The experiment showed that the R-LBP algorithm is capable of amplifying the texture features among citrus fruits at distinct ripeness stages. The YOLO-CIT model combined with the R-LBP algorithm has a Precision of 88.13%, a Recall of 93.16%, an F1 score of 90.89, a mAP@0.5 of 85.88%, and 6.1ms of average detection speed for citrus fruit ripeness identification in complex environments. The model demonstrates the capability to accurately and swiftly identify citrus fruits at distinct ripeness stages in real-world environments, effectively guiding the determination of picking targets and path planning for harvesting robots.

## 1 Introduction

Citrus fruits possess high nutritional and economic value and are widely cultivated globally (Wang et al., 2022). As the largest citrus-cultivating country, China currently relies predominantly on manual harvesting, leading to significant labor and time costs (Pei et al., 2022). In the context of Agriculture 4.0, the development of fruit-picking robots has emerged as a crucial research direction (Wang et al., 2022 ). The identification of distinct ripeness stages in citrus is instrumental in achieving intelligent sorting and harvesting, thereby enhancing orchard productivity and fruit quality (Sun et al., 2019).

During the ripening process of citrus fruits, the accumulation of sucrose leads to a reduction in chlorophyll and an increase in carotenoids. Consequently, the peel transitions from green to orange in color (Iglesias et al., 2001). Simultaneously, the synthesis of epicuticular wax on the fruit peel gradually occurs, increasing the smoothness of the peel (Romero and Lafuente, 2020). Based on its visual characteristics, citrus fruits with an orange area covering more than 80% of the total peel surface area are generally defined as ripe; otherwise, they are considered unripe. However, citrus fruits within the same orchard often exist at distinct ripeness stages (Gupta et al., 2021). Therefore, harvesting robots need to rapidly and accurately identify citrus fruits at distinct ripeness stages.

The color, shape, texture, and other features of the fruits are commonly used as criteria for ripeness identification. Image processing methods are widely applied in the field of citrus fruit ripeness identification. By combining the color difference map of citrus fruits under normal conditions with the brightness map under illumination, and utilizing color characteristics for threshold segmentation, Lu effectively solves the impact of illumination on citrus ripeness identification (Lu et al., 2014). Regarding the orange features in citrus images, which are predominantly manifested in the Cr channel of the YCbCr color space, Peng employed an improved fuzzy C-means clustering threshold segmentation method (FCM) to achieve accurate identification of ripe citrus fruits (Peng et al., 2014). Under complex weather conditions, the color features of citrus images are variable. Qiang utilized the morphological features of citrus fruits and employed a multi-class support vector machine based on morphological operations to effectively identify ripe citrus fruit (Qiang et al., 2014). Xu applied the Otsu adaptive thresholding method to the V component of the YUV color space. The Canny edge detection algorithm was employed to obtain the morphological features of citrus fruits, enabling a more accurate identification in the environment (Xu et al., 2020). Texture features are also among the essential characteristics of citrus fruits. In addressing the issue of green, unripe citrus fruits being close in color to the background, Zhao combined the results of the Adaptive Red-Blue color map (ARB) and Histogram Equalization for Hue (HEH). By utilizing five selected texture features to eliminate false positives, accurate identification of green, unripe citrus fruits was achieved (Zhao et al., 2016).

In recent years, deep learning models have been widely applied in harvesting robots, achieving high automation and effectively improving the accurate classification of complex ripeness features. Xiong proposed a Des-YOLOv3 network, which accurately identifies small and occluded citrus fruit targets (Xiong et al., 2020). Based on the YOLOv5s network, the BCAM (bidirectional cross attention mechanism) attention mechanism was added by Yang, resulting in enhanced identification accuracy for various fruits, including citrus (Yang et al., 2022). Regarding the differences in characteristics among citrus fruits at distinct ripeness stages, Lu utilized a Resnet backbone structure that integrates deep and shallow features to construct the Mask-Rcnn network. This approach precisely identified citrus fruits at distinct ripeness stages (Lu et al., 2022). For real-time identification of citrus fruits, Chen combined the Canopy algorithm and K-Means++ algorithm to automatically determine the input image size. Additionally, the Scientific Control of Pruning (SCOP) algorithm was applied to prune the YOLOv4 network, enabling real-time identification of citrus fruits (Chen et al., 2022). The improved deep learning model can effectively detect the maturity of citrus fruits.

Modern harvesting robots need to accurately identify unripe and ripe citrus fruits during the harvesting process, avoiding picking unripe ones and ensuring the precise harvesting of ripe ones (Yang et al., 2020). However, the green color of unripe citrus fruits is similar to the background color of leaves, resulting in significant detection omissions; Some partially unripe citrus fruits exhibit both orange and green characteristics, making it challenging to differentiate them from ripe citrus fruits with similar color features, leading to issues of misidentification (Lu et al., 2018). This study proposed an R-LBP (Roughness-Local Binary Pattern) algorithm, an improvement upon the LBP algorithm. By computing two sets of coefficients of variation in grayscale values in eight different directions around the current pixel, one including the grayscale value of the current pixel and the other excluding it, the R-LBP algorithm determines encoding based on the difference in the degree of variation between the two sets of coefficients. The citrus fruit images processed by this algorithm are added to the training dataset to better facilitate the learning of texture and morphological features of citrus fruits; Furthermore, a YOLO-CIT (You Only Look Once-Citrus) model, an improvement upon YOLOv5s, is proposed. The backbone network of this model combines the C3 model with the CBAM (Convolutional Block Attention Module) attention mechanism, leveraging both channel and spatial features to enhance the model’s capability to extract characteristics of distinct ripeness stages in citrus. In the neck network of the model, a Ghostnet structure is utilized, transforming some regular convolutions into linear mappings to reduce computational complexity and improve the model’s inference speed. Multiple experiments were conducted to verify the effectiveness of the proposed R-LBP algorithm and the performance of the YOLO-CIT model.

## 2 Materials and methods

### 2.1 R-LBP algorithm

**Figure 1:**
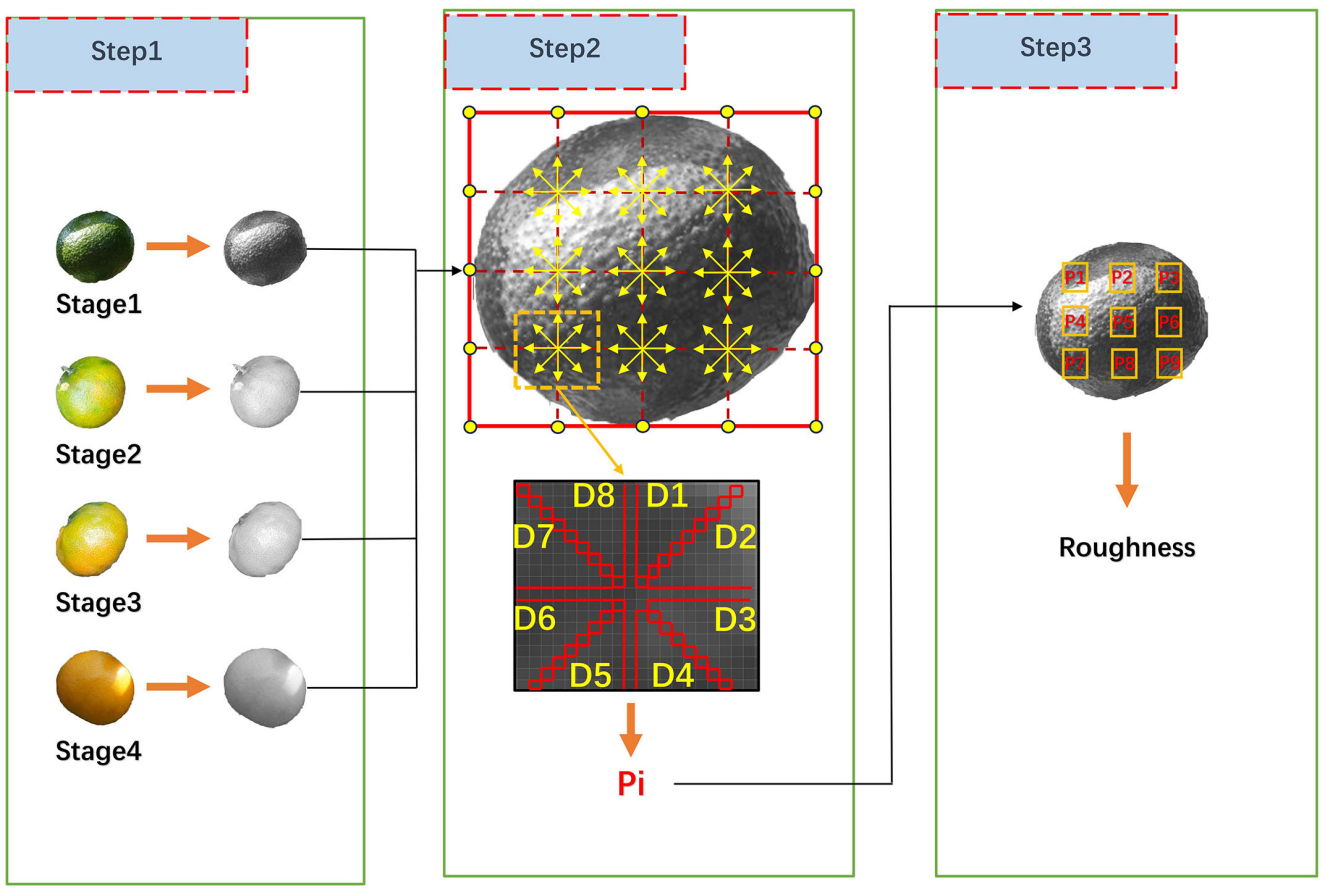
Calculation process for peel roughness of citrus fruits.

The LBP algorithm is a method that reflects the local texture features of an image by describing the texture variations around a pixel. Common texture features include roughness, directionality, contrast, and so on (Ojala et al., 2002). In the ripening process of citrus fruits, the synthesis of epicuticular wax leads to a noticeable change in the roughness of the peel. In digital images, the roughness of fruit peel can be represented by the degree of variation in pixel grayscale values. The calculation process of peel roughness for citrus fruits at distinct ripeness stages is illustrated in **Figure 1**.

In Step 1, citrus fruits at four distinct ripeness stages are sequentially selected and converted into grayscale images. In Step 2, for each grayscale image, the longer and shorter sides of its bounding rectangle are divided into four equal parts successively to obtain intersection points, defining nine sampling regions. In each sampling region, the average pixel difference in eight directions is calculated sequentially, as shown in Equations 1, 2.


(1) $$\begin{array}{c} D_{i}=\frac{\sum_{i=1}^{N}\left|X_{i}-X^{\prime }\right|}{N} \end{array}$$

(2) $$\begin{array}{c} P_{i}=\frac{\sum_{i=1}^{8}D_{i}}{8} \end{array}$$

Where $X_{i}$ represents the grayscale value of the pixel at position i , N represents the number of pixels in that direction, $X^{\prime }$ represents the average value of N pixels, and $D_{i}$ represents the mean difference of pixel grayscale values in that direction.

**Figure 2:**
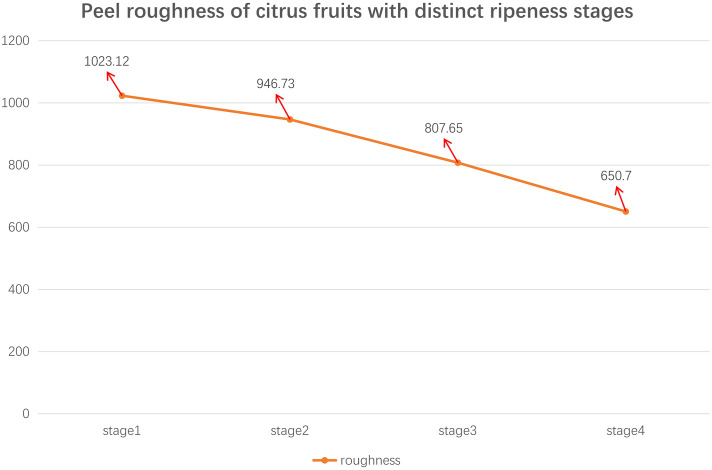
Peel roughness of citrus fruits with distinct ripeness stages.

In Step 3, the roughness of the fruit peel is obtained by calculating the average of the nine sampling regions. The results for distinct ripeness stages of the fruit are illustrated in **Figure 2**.

**Figure 3:**
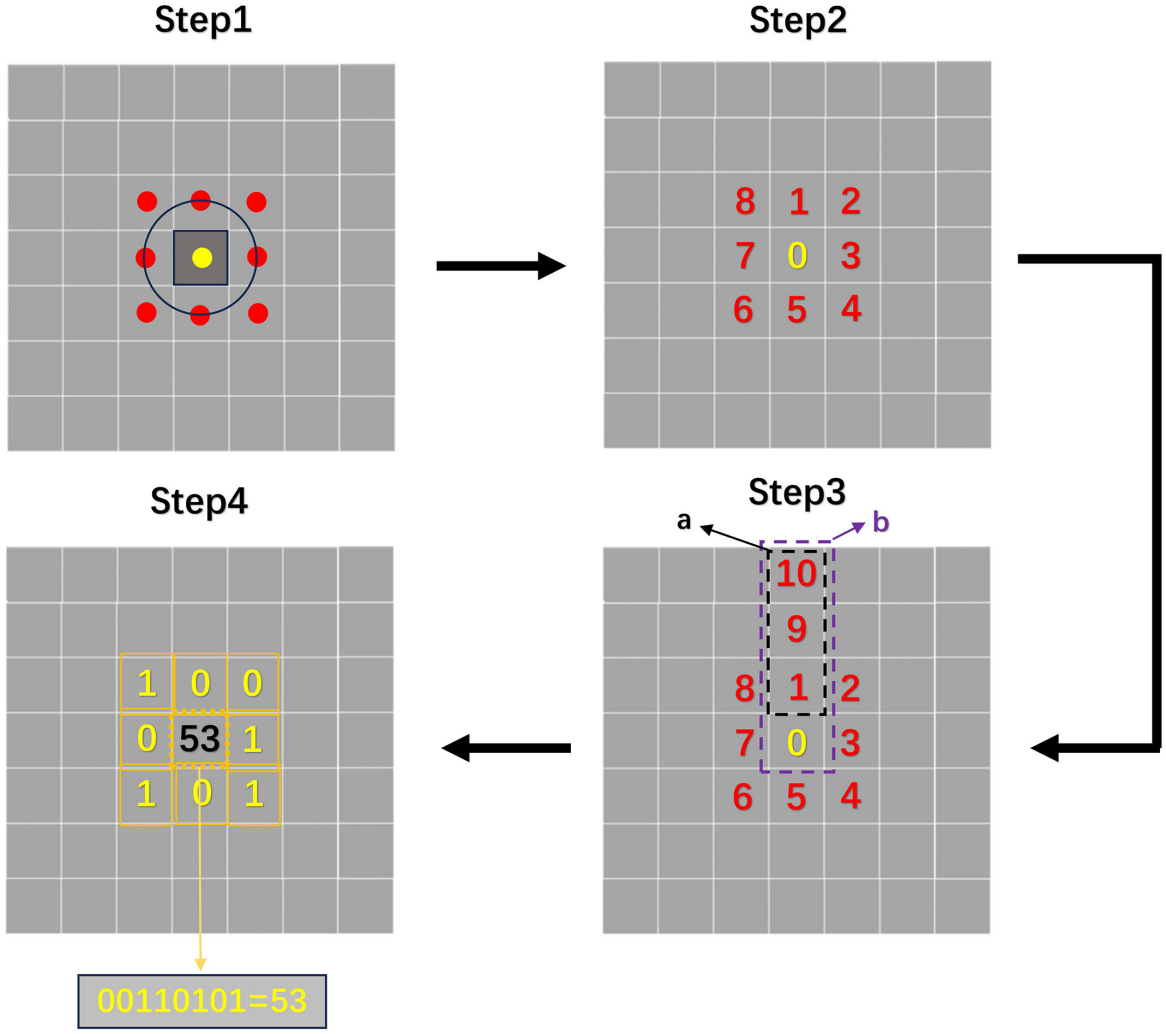
R-LBP algorithm process.

**Figure 2** shows that there is a decreasing trend in the roughness of citrus fruit peel as it matures. Based on this characteristic, the R-LBP algorithm, which utilizes peel roughness for encoding, is proposed. The method’s workflow is illustrated in **Figure 3**.

The R-LBP algorithm processes each pixel in the image sequentially to calculate its final grayscale value. In Step 1, the encoding relies on the eight pixels surrounding the target pixel, which are named reference pixels. In Step 2, the reference pixels are numbered sequentially in a clockwise order. In Step 3, The coefficient of variation reflects the fluctuation in a set of data, and the variation in pixel grayscale values can represent the roughness of the peel. Taking reference pixel 1 as an example, the coefficient of variation ( $CV_{a}$ ) is calculated for the array formed by pixels 1, 9, and 10 in its direction. Additionally, the coefficient of variation ( $CV_{b}$ ) is calculated for the array formed by adding the target pixel to the aforementioned array (pixels 0, 1, 9 and 10). The calculation process is shown in Equations 3, 4.


(3) $$\begin{array}{c} CV_{a}=\left(\frac{\sqrt{\frac{\sum_{i=2}^{4}{\left(X_{i}-X^{\prime }\right)}^{2}}{3}}}{X^{\prime }}\right)\times 100\% \end{array}$$

(4) $$\begin{array}{c} CV_{b}=\left(\frac{\sqrt{\frac{\sum_{i=1}^{4}{\left(X_{i}-X^{\prime }\right)}^{2}}{4}}}{X^{\prime }}\right)\times 100\% \end{array}$$

Where $X_{i}$ represents the grayscale value of the pixel at coordinate i , and $X^{\prime }$ represents the mean value within the array.

If the difference between $CV_{a}$ and $CV_{b}$ is greater than 15% of $CV_{a}$ , it indicates that the addition of the target pixel significantly affects the roughness between the existing reference pixels in that direction (Brown, 1998). Moreover, the texture features in that direction are strong. In such cases, the encoding of the current reference pixel is set to 1; otherwise, it is set to 0. The specific encoding process is shown in Equation 5.


(5) $$\begin{array}{c} \{\begin{array}{c} 1\;\frac{\left|CV_{a}-CV_{b}\right|}{CV_{a}}>15\%\\ \;0\;\frac{\left|CV_{a}-CV_{b}\right|}{CV_{a}}\leq 15\% \end{array} \end{array}$$

In Step 4, the encoding results from the eight reference pixels are combined in the order of their numbering to obtain a binary outcome. This binary result is then converted to decimal and serves as the new grayscale value for the current pixel. The same process is applied to other pixels, and the final result of the R-LBP algorithm processing is obtained.

### 2.2 Dataset construction of citrus images

#### 2.2.1 Image acquisition of citrus data

There are citrus orchards located in Zengcheng District, Guangzhou City, Guangdong Province, China (23°16′N, 113°51′E) for dataset collection. The Canon 200D Mark II DSLR camera, equipped with an 18-55mm lens set to fully automatic mode, is used for capturing images of citrus fruits. Take images every two days, a total of 5 times, from 10 am to 12 am. A total of 1533 raw image data were collected, and saved in.jpeg format, with a resolution of 4032×3024 pixels. The collection information for the initial data is presented in **Table 1**.

**Table 1:** Initial dataset composition table.

Order	Date	Weather	Num
1	2022/10/09	Cloudy	297
2	2022/10/11	Sunny	305
3	2022/10/13	Sunny	312
4	2022/10/15	Sunny	285
5	2022/10/17	Cloudy	334

**Figure 4:**
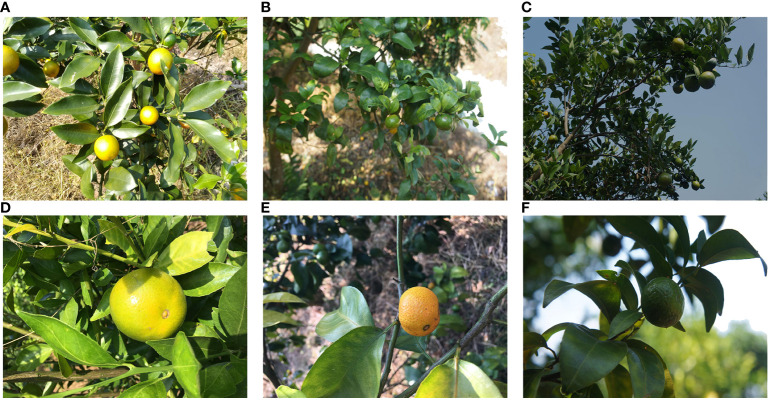
Sample image of initial dataset: **(A)** Medium distance exposure citrus image; **(B)** Medium range natural light citrus image; **(C)** Medium distance backlight citrus image; **(D)** Close range exposure citrus image; **(E)** Close range natural light citrus image; **(F)** Close range backlight citrus image.

The collected data underwent preliminary screening, removing images with out-of-focus, motion blur, or severe distortion. Some images from the initial dataset were augmented through random flips, tilts, and other operations. The initial dataset can be divided into several categories based on lighting intensity and shooting distance, as shown in **Figure 4**. The specific composition is detailed in **Supplementary Table 2**.

#### 2.2.2 R-LBP–based citrus images texture enhancement

**Figure 5:**
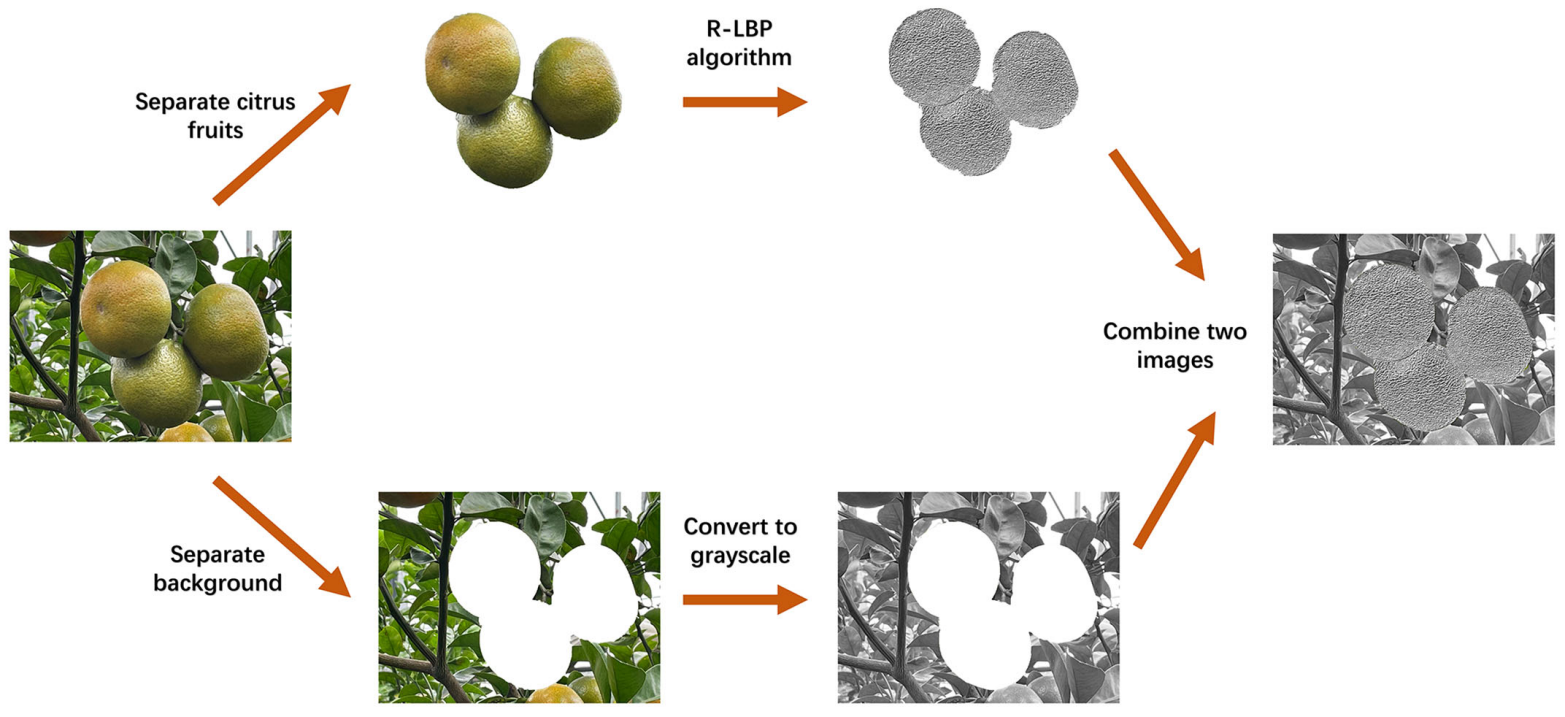
Image synthesis process.

The R-LBP algorithm primarily processes the fruit segment of citrus images. Therefore, in this study, artificially synthesized images were obtained to enhance the texture features of the fruit segment at distinct ripeness stages in citrus images. The synthesized images are added to the training dataset to enhance the features that the model can learn. The processing workflow for artificially synthesized images is illustrated in **Figure 5**.

By using Photoshop, the citrus fruit area and background area are separately extracted. The citrus fruit undergoes processing with the R-LBP algorithm to enhance its texture features. The background region is converted to a grayscale image to remove its color characteristics. The two processed results are then combined to create the final image.

**Figure 6:**
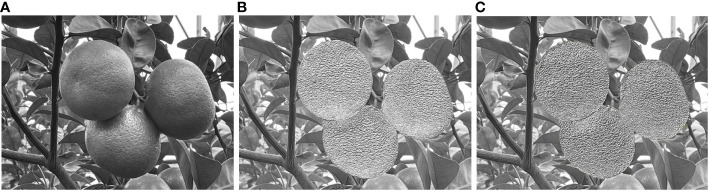
Sample image of additional dataset: **(A)** Grayscale citrus image; **(B)** Synthetic citrus images based on LBP; **(C)** Synthetic citrus images based on R-LBP.

The additional training set consists of synthesized images based on close-range citrus images with clear texture features. It is used to test the effect of adding images processed by the R-LBP algorithm to the training set on model performance. The sample images of the additional training set are shown in **Figure 6**, and the specific composition of the additional dataset is shown in **Supplementary Table 1**.

### 2.3 Construction of YOLO-CIT model

#### 2.3.1 Backbone network of the YOLO-CIT model

**Figure 7:**
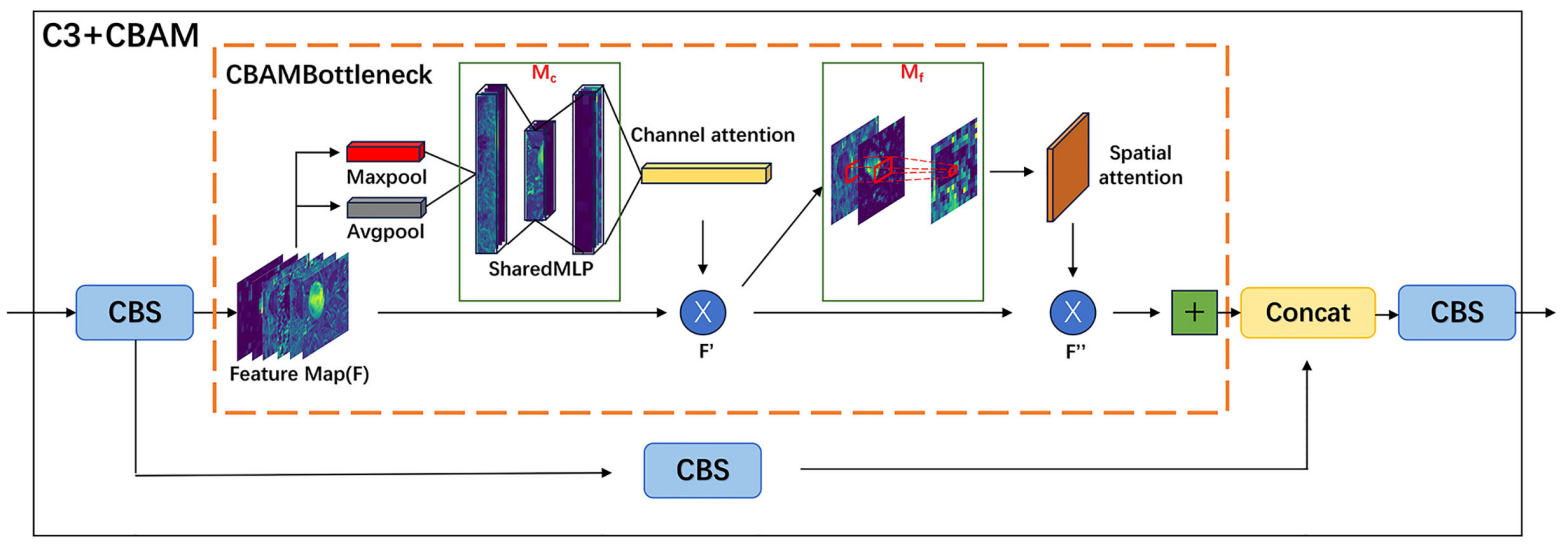
C3+CBAM module calculation process.

The YOLO-CIT model proposed in this paper is built upon the YOLOv5s model. The backbone network structure of the YOLO-CIT model is formed by combining the CBAM attention mechanism with the C3 module. The computational process is illustrated in **Figure 7**.

The feature maps are entered into the CBAM Bottleneck, where maximum pooling and average pooling features are computed. These two features are then input into a Multi-Layer Perceptron (MLP), and channel attention is calculated. The computation process is depicted in Equation 6.


(6) $$\begin{array}{c} M_{c}\left(F\right)=\sigma \left(W_{1}\left(W_{0}\left(F_{avg}^{c}\right)\right)+W_{1}\left(W_{0}\left(F_{max}^{c}\right)\right)\right) \end{array}$$

Where σ represents the sigmoid function, $W_{0}\in R^{c/r*c}$ and $W_{1}\in R^{c*c/r}$ represent the shared weights of the MLP, r represents the reduction ratio, c represents the length of the feature map, $F_{avg}^{c}$ represents the average pooling feature, and $F_{max}^{c}$ represents the maximum pooling feature.

Channel attention and feature maps are broadcasted, and the intermediate feature output F ‘is calculated, as shown in Equation 7.


(7) $$\begin{array}{c} F^{\prime }=M_{c}\left(F\right)⊗F \end{array}$$

Average pooling and maximum pooling operations are applied along the channel axis of the middle feature map, the two results are connected through standard convolutional layers and spatial attention is generated. The calculation process is shown in Equation 8.


(8) $$\begin{array}{c} M_{s}\left(F\right)=\sigma \left(f^{7\times 7}\left(\left[F_{avg}^{s};F_{max}^{s}\right]\right)\right) \end{array}$$

Where $F_{avg}^{s}$ represents the two-dimensional average pooling feature, $F_{max}^{s}$ represents the two-dimensional maximum pooling feature, and $f^{7*7}$ represents the convolution operation with a filter size of 77.

Spatial attention further aggregates with the middle feature map through broadcasting, and the final output $F^{\prime \prime }$ of the module is calculated, as shown in Equation 9.


(9) $$\begin{array}{c} F^{\prime \prime }=M_{s}\left(F^{\prime }\right)⊗F^{\prime } \end{array}$$

#### 2.3.2 Neck network of the YOLO-CIT model

**Figure 8:**
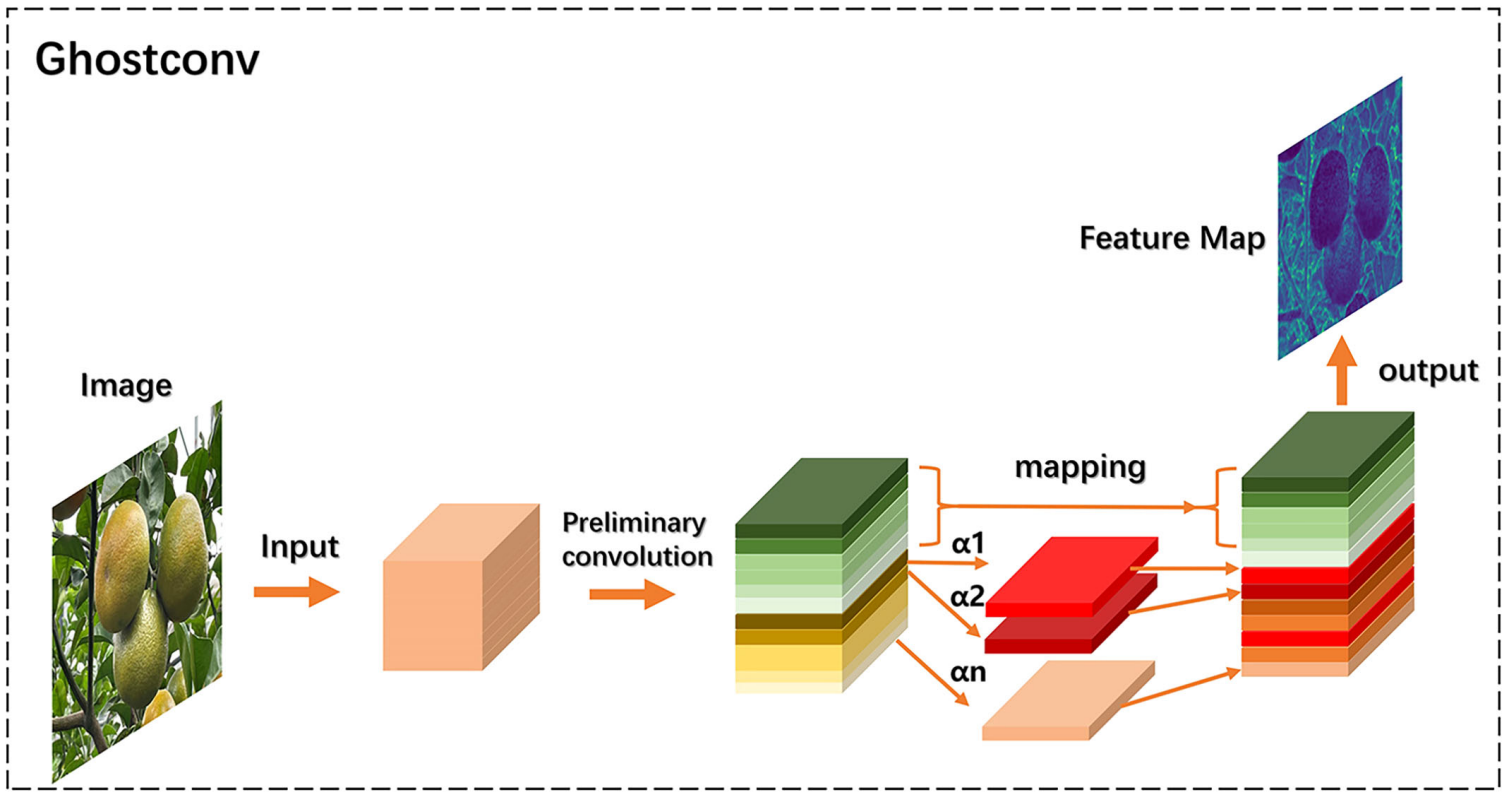
Ghostconv module calculation process.

In the neck network of the YOLO-CIT model, Ghostconv is used instead of regular convolution. The structure of Ghostconv for processing input content is shown in **Figure 8**.

In the context of citrus fruit image processing, the initial features of the input image are extracted through an initial convolution process using convolution kernels. The outcomes of the initial convolution are divided into two segments, where one segment undergoes mapping, and the other segment, including α1, α2, …, αn initial convolutional feature maps, undergoes additional convolutional operations. The outputs from these two segments are then merged to generate the final output feature map.

#### 2.3.3 YOLO-CIT model

**Figure 9:**
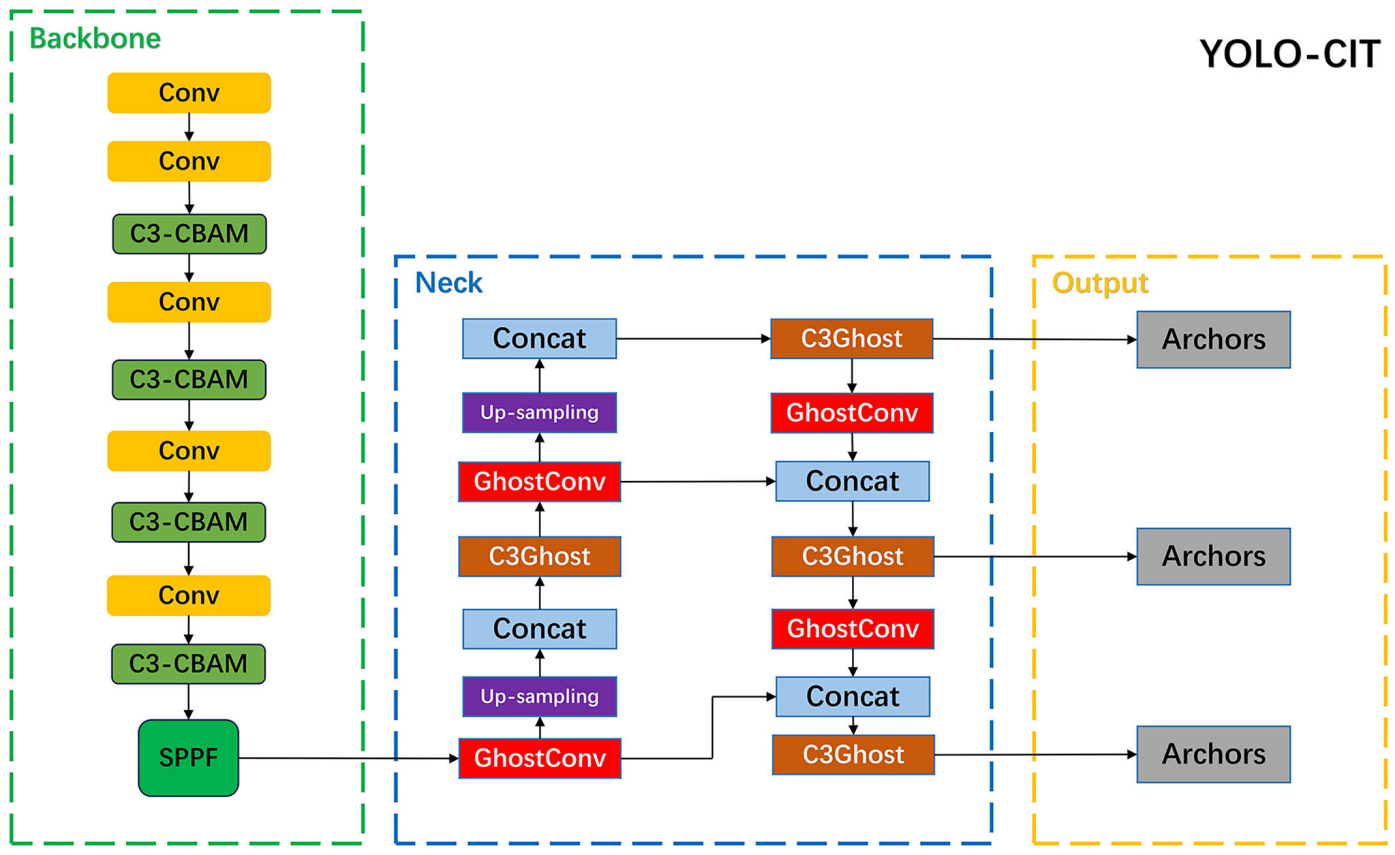
YOLO-CIT Network Architecture.

The specific composition structure of the YOLO-CIT network is shown in **Figure 9**. The initial image of citrus fruit is input into the backbone network and processed by modules such as C3-CBAM to generate a feature map that combines channel attention and spatial attention; The feature map is further input into the neck network, and through lightweight convolution and up sampling operations in the GhostConv module, a prediction box is generated. Citrus fruits with distinct ripeness stages are identified.

### 2.4 Experiments

To validate the effectiveness of the YOLO-CIT model and R-LBP algorithm in this study, three sets of experiments were conducted sequentially.

In the first set of experiments, 200 close-range images of citrus fruits were selected. These images were divided into 50 groups, with each group containing four images representing distinct ripeness stages of the citrus fruit. Various algorithms, including grayscale processing, LBP algorithm, and R-LBP algorithm, were applied to the fruit segment of the citrus fruit images respectively. The difference in surface roughness, as introduced in Section 2.1.1, was used to evaluate the enhancement effects of different algorithms on surface roughness. The average difference in roughness values between adjacent stages within each group of images after processing by different algorithms was recorded and analyzed.

In the second set of experiments, the YOLO-CIT model was trained on the basic training set, supplemented with additional datasets processed using grayscale conversion, LBP algorithm, and R-LBP algorithm respectively. The performance parameters of the model were recorded and analyzed.

In the third set of experiments, the YOLO-CIT model proposed in this study, along with several common deep learning network models such as models of YOLOv4, YOLOv5s, YOLOv7, YOLOX, YOLOv8s, and faster-RCNN, was trained using the basic dataset. A comparative performance analysis of the models was conducted.

The experimental hardware setup primarily involves a computer system featuring an Intel i5-13600kf processor, 32 GB RAM, and a GeForce GTX 4080 GPU. The computer is configured with CUDA 11.2 parallel computing architecture and utilizes the NVIDIA cuDNN 8.0.5 GPU acceleration library. The software simulation environment is built on the PyTorch deep learning framework (Python version 3.10). The data pre-processing involved the utilization of Labeling, Photoshop 2018, and Matlab2020b. For configuring and managing the virtual environment, Anaconda was employed, and program compilation and execution were carried out using Pycharm. Model performance metrics mainly include P (precision), R (recall), F1 (harmonic average), AP (average precision), and mAP@0.5 (mean average precision) shown in Equations 10–14.


(10) $$\begin{array}{c} precision=\frac{T_{p}}{T_{p}\;+\;F_{p}} \end{array}$$

(11) $$\begin{array}{c} recall=\frac{T_{p}}{T_{p}\;+\;F_{N}} \end{array}$$

(12) $$\begin{array}{c} F_{1}=\frac{2\;\times \;precision\;\times \;recall}{precision\;+\;recall} \end{array}$$

(13) $$\begin{array}{c} AP=\frac{\sum^{precision}}{N} \end{array}$$

(14) $$\begin{array}{c} mAP@0.5=\frac{\sum_{i=1}^{K}AP_{i}}{N_{C}} \end{array}$$

where $T_{p}$ represents the number of citrus fruits correctly identified, $F_{p}$ represents the number of citrus fruits incorrectly identified, $F_{N}$ represents the number of missed citrus fruits, N represents the total number of images, and $N_{C}$ represents the number of categories of citrus fruit ripeness stages. AP representing the integral of accuracy rate to recall rate is equal to the area under the P-R curve. mAP@0.5 is the average of the average precision of all categories.

## 3 Results

### 3.1 Performance analysis of citrus fruit texture enhancement

**Figure 10:**
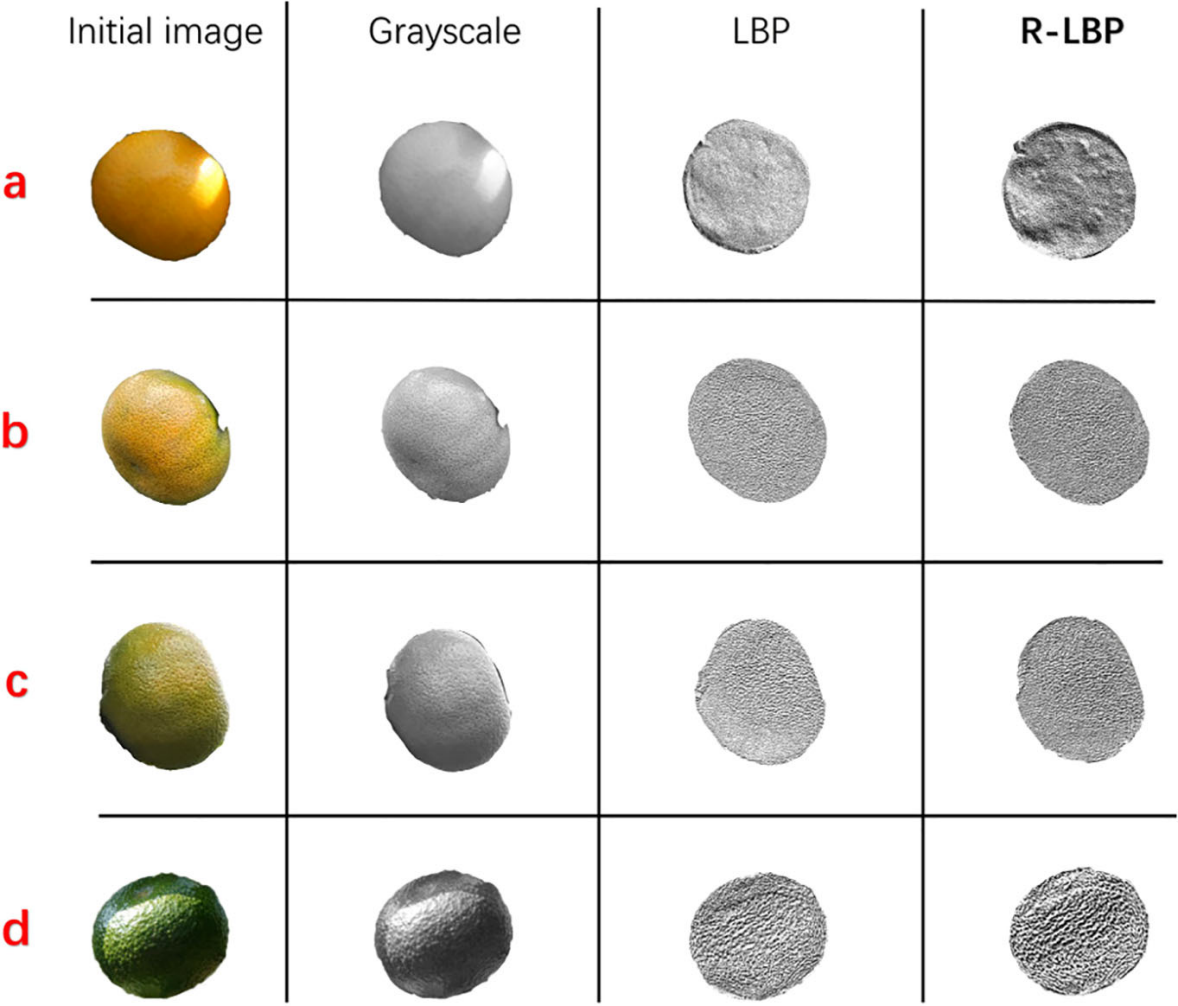
**(A–D)** Different ripening stages of citrus fruits.

**Figure 11:**
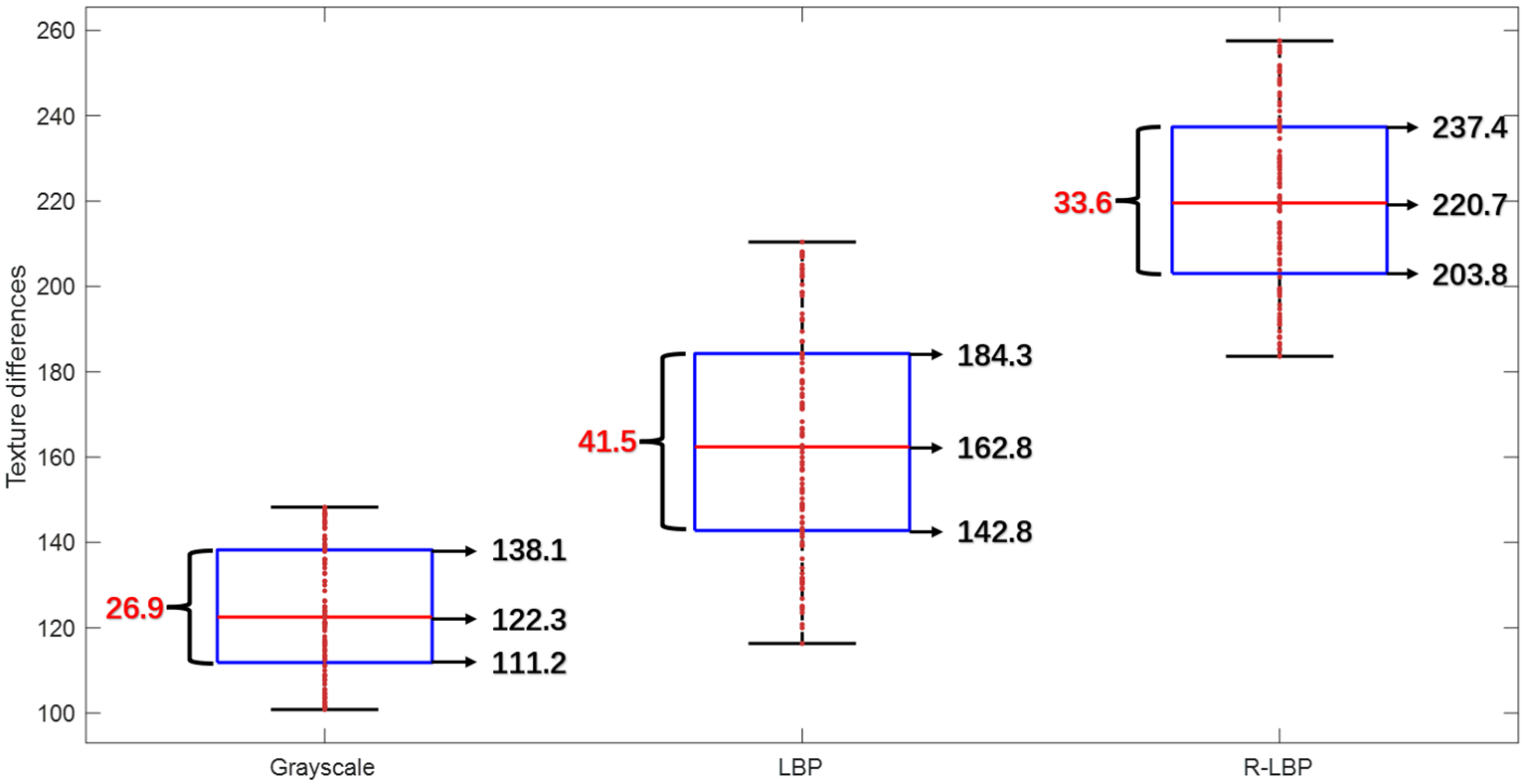
Citrus fruit epidermal roughness difference across ripening stages.

In the first set of experiments, distinct ripeness stages of citrus fruits were processed using grayscale conversion, LBP algorithm, and R-LBP algorithm respectively, as shown in **Figure 10**. The differences in peel roughness between citrus fruits at different maturity stages are depicted in **Figure 11**.

The median in peel roughness difference of images processed by grayscale conversion is 122.3, with an upper quartile of 138.1, a lower quartile of 111.2, and an interquartile range of 26.9. The dispersion of peel roughness in different images is relatively small; The median in peel roughness difference of images processed with the LBP algorithm is 162.8, with an upper quartile of 184.3, lower quartile of 142.8, and an interquartile range of 41.5. The results show a higher level of dispersion among different images in this case. The median peel roughness difference of images processed with the R-LBP algorithm is 220.7, which is respectively 98.4 and 57.9 higher than the results obtained with the grayscale conversion and LBP algorithm. The interquartile range of peel roughness for images processed with the R-LBP algorithm is 33.6, which is 6.7 higher than the grayscale conversion and 7.9 smaller than the LBP algorithm. The data dispersion is relatively stable. Images processed with the R-LBP algorithm exhibit more distinct and relatively stable texture features compared to images processed with grayscale conversion and the LBP algorithm, which are useful for distinguishing distinct ripeness stages of citrus fruits.

### 3.2 Comparison of identification performance of YOLO-CIT model trained with different datasets

**Figure 12:**
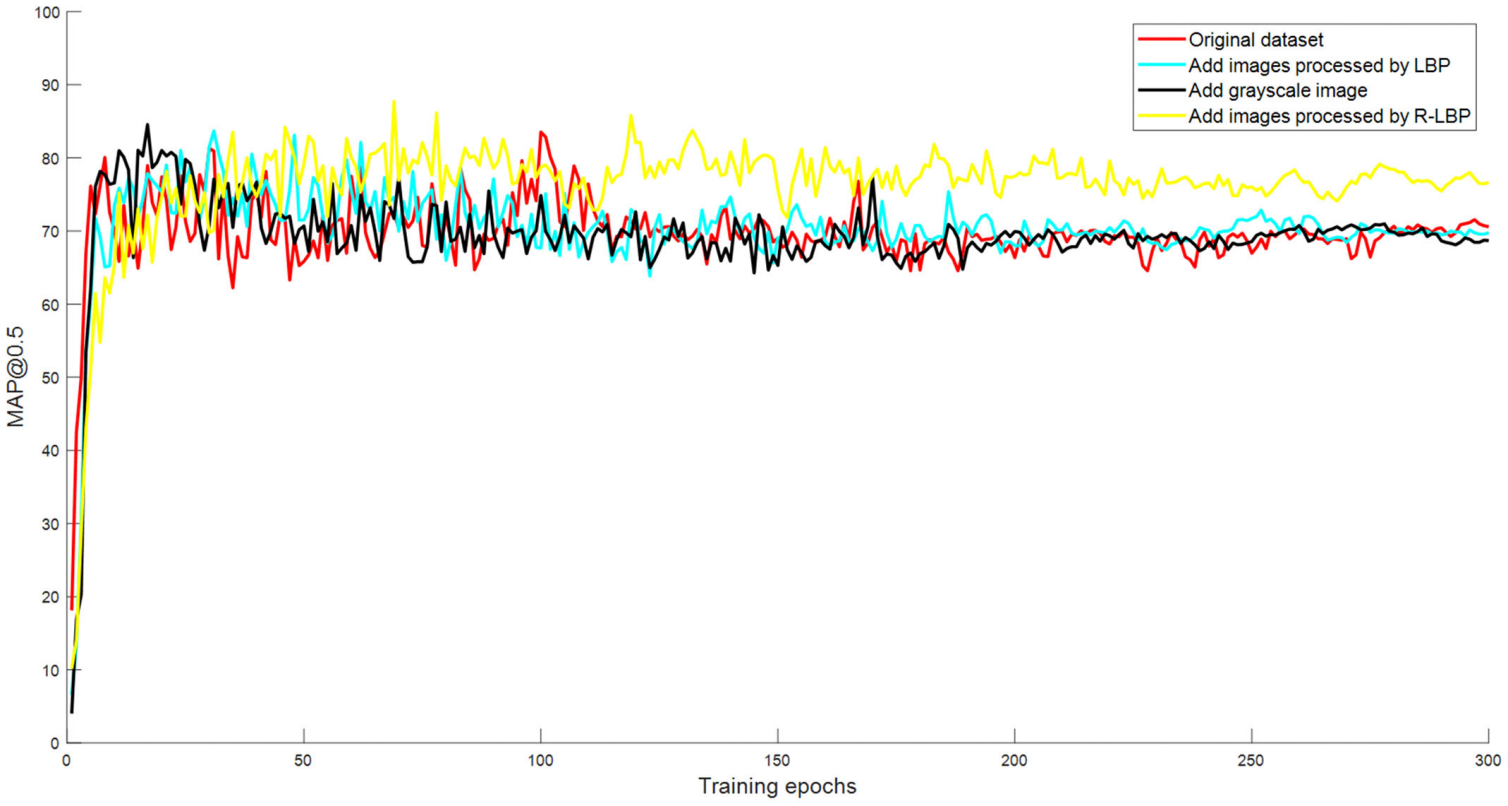
Change in YOLO-CIT model’s mAP@0.5 trained on different datasets.

In the second set of experiments, the YOLO-CIT model was trained using the same base dataset but with different additional datasets. The variation of map@0.5 during the training process is illustrated in **Figure 12**, and the performance parameters of the model are listed in **Table 2**.

**Table 2:** Performance of models trained on different datasets.

dataset	mAP@0.5	Precision/%	Recall/%	F1score
Original dataset	83.52	87.50	90.01	88.74
Add grayscale images	84.17	86.42	91.31	88.79
Add LBP images	83.39	88.70	91.63	90.14
Add R-LBP images	85.88	88.73	93.16	90.89

By observing **Figure 12**, it can be seen that the four curves exhibit significant differences during the training process mAP@0.5 The value rapidly increases and remains relatively stable after the epoch reaches 100. Among them, when the model uses the additional dataset with R-LBP or LBP, the fluctuation of the curve is smaller, and the mAP@0.5 value is more stable. The YOLO-CIT model trained with the R-LBP additional dataset exhibits a higher stable mAP@0.5 curve, indicating the best detection accuracy among the curves. According to **Table 2**, the YOLO-CIT model trained with the R-LBP additional dataset achieves mAP@0.5 values that are 2.36, 1.71, and 2.49 higher than the YOLO-CIT models trained with the base dataset, additional grayscale image dataset, and additional LBP dataset, respectively. Its Precision is slightly higher than other results, and its Recall is approximately 2% higher than the others. The F1 score of the YOLO-CIT model trained with the additional R-LBP dataset is 2.15, 2.1, and 0.75 higher than the other results, respectively. The YOLO-CIT model trained with the additional R-LBP dataset exhibits the best overall performance.

### 3.3 Performance comparison among various network models

**Figure 13:**
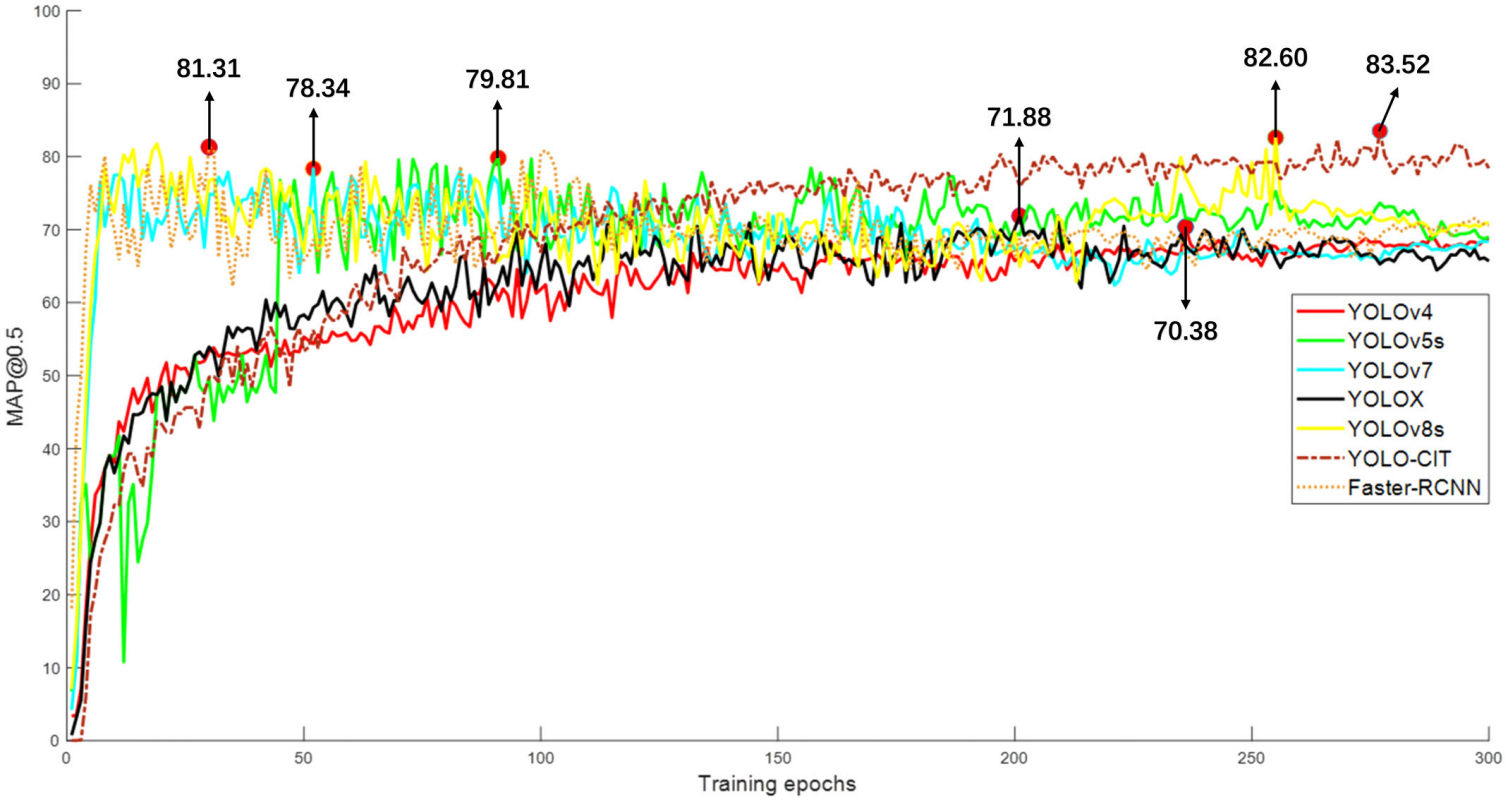
Variation of mAP@0.5 during training across different models.

In the third set of experiments, different deep-learning models were trained using the base dataset. The variation of mAP@0.5 during the training process is illustrated in **Figure 13**, and the model performance parameters are shown in **Table 3**.

**Table 3:** Performance of models trained on different datasets.

dataset	mAP@0.5	Precision/%	Recall/%	F1score
YOLOv4	70.83	76.89	81.02	78.90
YOLOv5s	79.81	86.10	85.19	85.64
YOLOv7	78.34	85.82	82.33	84.04
YOLOX	71.88	76.21	78.04	77.11
YOLOv8s	82.60	86.15	85.67	85.91
YOLO-CIT	83.52	87.50	90.01	88.73
Faster-RCNN	81.31	87.01	87.93	87.47

**Figure 13** shows that the mAP@0.5 curves of YOLO-CIT, YOLOX, and YOLOv4 models gradually increase during the training process and tend to stabilize after reaching epoch 150. This indicates that the models can effectively learn features of citrus fruits at distinct ripeness stages, resulting in a relatively stable improvement in accuracy in citrus fruit identification. The mAP@0.5 curves of the YOLOv5s, YOLOv7, YOLOv8s, and Faster-RCNN models show a rapid initial rise during training. However, between epochs 100 and 150, there is a declining trend, and after reaching epoch 150, there is a noticeable fluctuation. This indicates that these models exhibit differences in feature learning during the training process, leading to temporary decreases in detection accuracy and insufficient stability in the models. The YOLO-CIT model achieved its best mAP@0.5 value of 83.52 during training, significantly surpassing the mAP@0.5 values of the YOLOv4 and YOLOX models, and slightly outperforming the YOLOv5s, YOLOv7, YOLOv8s, and Faster-RCNN models. The YOLO-CIT model exhibits the highest detection accuracy. Observing **Table 3**, it can be seen that the YOLO-CIT model has Precision values higher than other models by 0.49% to 11.29%, Recall values higher by 2.08% to 11.97%, and F1 scores higher by 1.26 to 11.62. The YOLO-CIT model demonstrates the best performance.

**Figure 14:**
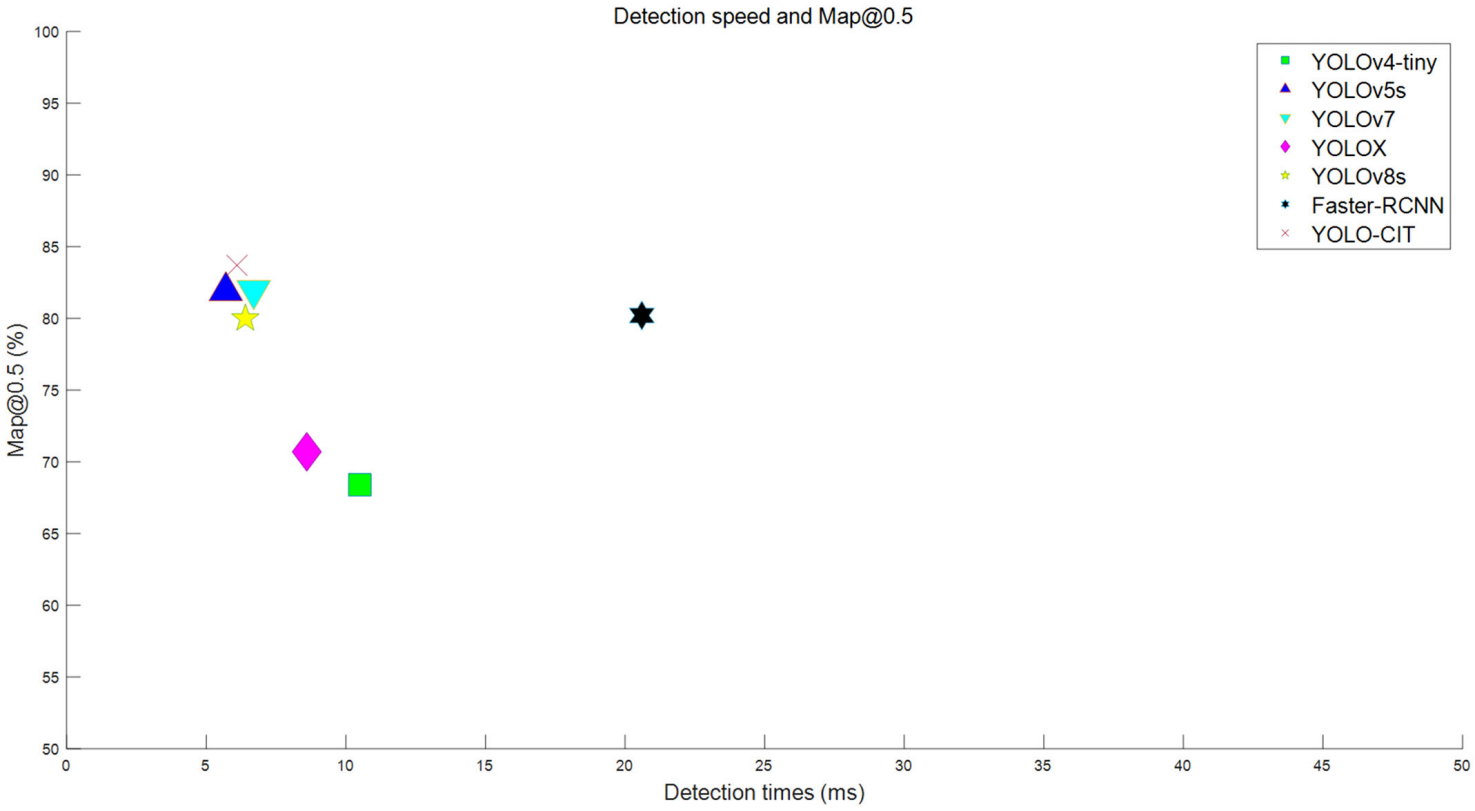
Detection time and mAP@0.5 across various models.

The average detection time of the YOLO-CIT model compared to other experimental models map@0.5 The distribution is shown in **Figure 14**.

According to **Figure 14**, the YOLO-CIT, YOLOv5s, YOLOv7, and YOLOv8s models exhibit high average detection accuracy while maintaining a fast detection speed. The average detection speed for these models ranges from 5ms to 7ms, meeting the requirements for real-time detection. The detection speed of the YOLO-CIT model is slightly lower than that of the YOLOv5s, but it achieves higher average detection accuracy than the YOLOv5s model.

**Figure 15:**
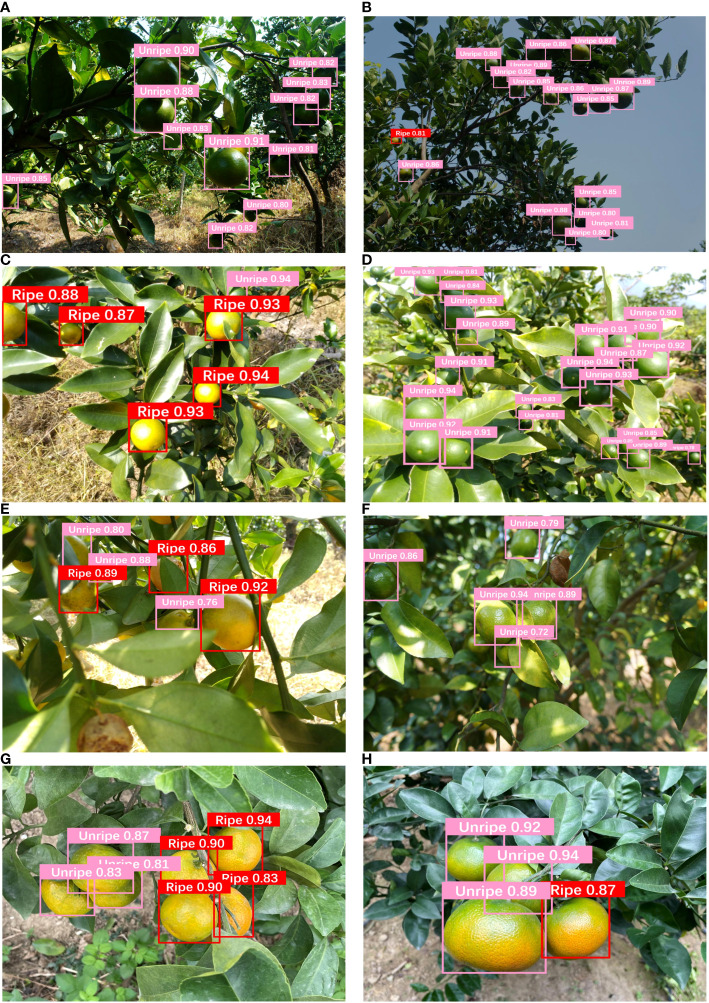
YOLO-CIT model: citrus ripeness identification in varied environments: **(A, B)** Backlight environment; **(C, D)** Exposure environment; **(E, F)** The situation where leaves cover the fruit; **(G, H)** The dense distribution of citrus fruits.

To validate the ripeness identification capability of the YOLO-CIT model in real-world environments, the model was trained using the base dataset and an additional dataset processed with R-LBP. The trained model was then utilized to identify citrus fruit ripeness stages in diverse environmental conditions, encompassing both images and videos. Results are shown in **Figure 15** and **Table 4**.

**Table 4:** Performance of the model on video detection.

Model	Device	Accuracy/%	Video processing time/sec	FPS
YOLO-CIT	GPU(RTX-4080super)	86.54	4.51	76.36
	CPU(i5-13600KF)	86.17	30.02	11.47

According to **Figure 15**, it can be noted that YOLO-CIT accurately identifies the ripeness stages of citrus fruits under varying lighting conditions. For citrus fruits exposed to strong light or significant shadow coverage, the identification confidence reaches 0.8 or higher. In cases of dense growth or occlusion of citrus fruits, for those close and with complete shapes, the confidence in ripeness identification ranges from 0.88 to 0.94. For those farther away with incomplete shapes, the recognition confidence is above 0.76. The above results show that the YOLO-CIT model is capable of accurately and confidently identifying the ripeness of citrus fruits within the robot’s picking range under various environmental conditions. According to **Table 4**, it can be seen that the YOLO-CIT model achieves an accuracy of 86.54% and an FPS of 76.36 when detecting videos on GPU devices. Beyaz & Gül deployed a YOLOv4-tiny model similar to this one on the NVIDIA Jetson TX2 AI board, achieving an FPS of over 12 during the detection process (Beyaz and Gül, 2023). The parameter count of the YOLO-CIT model is smaller than that of the YOLOv4-tiny model. Applying this model to the same type of AI board can also achieve similar performance. It has a fast processing speed and can effectively connect cameras for real-time detection tasks.

## 4 Discussion

The green unripe citrus fruits have a similar color to the background, making it challenging to identify them using color features alone. This study additionally investigates from the perspective of texture features. The R-LBP algorithm proposed in Experiment 1 effectively amplifies the differences in peel roughness of citrus fruits at different ripeness stages. This increases the feature disparity between green citrus fruits and the green background, significantly improving the accuracy of identifying green citrus fruits and reducing instances of missed recognition. At the same time, it also enhances the recognition ability of the model for citrus fruits with different maturities. Enhancing the differences in features between various identification targets is beneficial for target identification, a conclusion that aligns with findings in other research (Sharan et al., 2013).

In Experiment 2, different types of additional datasets were added to the basic training set to train the YOLO-CIT model. The performance of the model trained with added grayscale and LBP images did not show improvement and even slightly decreased. For grayscale images, this may be because the color features of grayscale images interfere with the model’s feature learning, leading to a decrease in recognition accuracy. For LBP images, this may be because the texture features provided by the images processed by LBP are not sufficient to improve the performance of the model. On the other hand, the model trained with the additional dataset using the R-LBP algorithm exhibited improved performance. This improvement is attributed to the fact that the training set, while containing color images providing color features, also includes additional images that offer roughness texture features. This enhancement contributes to the model’s learning effectiveness. Including samples in the training set that possess characteristics relevant to the application domain can effectively improve identification performance. Similar conclusions can be found in related studies (Liu, 2020; Han et al., 2021).

In Experiment 3, the YOLO-CIT model demonstrated the best Precision and mAP@0.5, indicating its ability to accurately identify citrus fruits at distinct ripening stages. The model also exhibited the best Recall, suggesting that it has a lower tendency to miss detections, providing comprehensive detection of the ripeness of all citrus fruits within the images. Due to the clear citrus color and texture features in the training set, the YOLO-CIT model incorporates the CBAM attention mechanism into the C3 module. The structure of C3 and CBAM uses more computation to extract texture features of citrus. The computational complexity is focused on the backbone network to learn the texture features. In the model’s neck, where the feature maps have already been initially formed, a lightweight Ghostconv module is utilized to reduce computational complexity. This not only avoids a loss in identification accuracy but also enhances the model’s detection speed. The above experiments concluded that introducing attention mechanisms in the backbone network while reducing computational complexity in the neck network can enhance the overall performance of the model. Similar conclusions can also be found in other research (McCool et al., 2017; Xu et al., 2023). In the identification results of citrus ripeness in different environments, the model accurately identifies citrus fruits at distinct ripeness stages. When the YOLO-CIT model is applied to GPU devices, its FPS exceeds 60 and detection accuracy exceeds 80%, indicating that the improved model can be combined with high frame rate cameras to provide real-time position information of different detection targets (Fang et al., 2019; Gündüz and Işık, 2023). It can be effectively applied to citrus harvesting robots, laying the foundation for their efficient harvesting operations. This aids in guiding the harvesting robot to avoid unripe citrus fruits, facilitating subsequent tasks in path planning for harvesting ripe citrus fruits (Ning et al., 2022; Yi et al., 2024). There were instances in the experimental results where some citrus fruits at a distance were not identified. These fruits were located beyond the operational range of the harvesting robot, rendering their ripeness identification irrelevant, and therefore, they can be disregarded.

## 5 Conclusion

This article first proposes an improved R-LBP algorithm based on LBP, which can amplify the peel roughness characteristics of citrus fruits with distinct ripeness stages. The synthesized images processed by the R-LBP algorithm are added to the training set, which can improve the identification accuracy of the model for citrus fruits with distinct ripeness stages. This article also proposes an improved YOLO-CIT model based on YOLOv5s, which can accurately and comprehensively identifies the ripeness stages of citrus fruits in complex environments, The specific conclusion is as follows:

1. An R-LBP algorithm based LBP, is proposed. This algorithm utilizes the grayscale value coefficient of variation for encoding, enhancing the differentiation in peel roughness among citrus fruits at distinct ripeness stages.
2. The fruit segment of citrus images is processed using the R-LBP algorithm, while the background is subjected to grayscale conversion to create synthetic images. Adding these images to the base training set enhances the model’s performance, effectively improving the accuracy of identifying green citrus fruits against a green background. Simultaneously, it reduces the misidentification rate for partially green and partially orange unripe citrus fruits.
3. The backbone network of the model is constructed using the C3+CBAM structure, and the traditional convolution in the neck network is replaced by Ghostconv. Thus, the YOLO-CIT model is established. The YOLO-CIT model, trained using the base dataset combined with the additional dataset processed with R-LBP, achieves a Precision of 88.13%, Recall of 93.16%, F1score of 90.89, and mAP@0.5 of 85.88%. It demonstrates comprehensive identification of the ripeness stages of citrus fruits in complex environments, including exposure, backlight, and occlusion.

These findings validate that the proposed YOLO-CIT model, in conjunction with the R-LBP algorithm, can comprehensively and accurately identify citrus fruits at distinct ripeness stages in complex environments. This provides accurate data for obtaining target coordinates and robotic arm parameters for the fruit-picking robot. In the future, we will study a harvesting path planning algorithm that comprehensively considers both mature and immature fruits, avoiding rotten citrus fruits and jointly planning the harvesting path.

## Data availability statement

The datasets presented in this study can be found in online repositories. The names of the repository/repositories and accession number(s) can be found in the article/**Supplementary Material**.

## Author contributions

CW: Conceptualization, Data curation, Investigation, Resources, Writing – review & editing. QH: Formal analysis, Methodology, Software, Writing – original draft, Writing – review & editing. CL: Data curation, Validation, Writing – review & editing. TZ: Funding acquisition, Project administration, Writing – review & editing. XZ: Funding acquisition, Project administration, Writing – review & editing.

## References

[B1] Beyaz A. Gül V. (2023). YOLOv4 and tiny YOLOv4 based forage crop detection with an artificial intelligence board. Braz. Arch. Biol. Technology. 66, e23220803. doi: 10.1590/1678-4324-2023220803

[B2] Brown C. (1998). “Coefficient of variation,” in Applied Multivariate Statistics in Geohydrology and Related Sciences, 155–157. doi: 10.1007/978-3-642-80328-4

[B3] Chen W. Lu S. Liu B. Chen M. Li G. Qian T. (2022). CitrusYOLO: A algorithm for citrus detection under orchard environment based on YOLOv4. Multimed. Tools Appl. 81, 31363–31389. doi: 10.1007/s11042-022-12687-5

[B4] Fang W. Wang L. Ren P. (2019). Tinier-YOLO: A real-time object detection method for constrained environments. IEEE Access 8, 1935–1944. doi: 10.1109/Access.6287639

[B5] Gündüz M.Ş. Işık G. (2023). A new YOLO-based method for real-time crowd detection from video and performance analysis of YOLO models. J. Real-Time Image Process. 20, 5. doi: 10.1007/s11554-023-01276-w 36744218 PMC9885395

[B6] Gupta A. Pathak U. Tongbram T. Medhi M. Terdwongworakul A. Magwaza L.. (2021). Emerging approaches to determine maturity of citrus fruit. Crit. Rev. Food Sci. Nutr. 62, 5245–5266. doi: 10.1080/10408398.2021.1883547 33583257

[B7] Han E. Smith A. Kemper R. White R. Kirkegaard J. Thorup-Kristensen K.. (2021). Digging roots is easier with AI. J. Exp. botany. 72, 4680–4690. doi: 10.1093/jxb/erab174 33884416

[B8] Iglesias D. Tadeo F. Legaz F. Primo-millo E. Talón M. (2001). *In vivo* sucrose stimulation of colour change in citrus fruit epicarps: Interactions between nutritional and hormonal signals. Physiologia plantarum. 112, 244–250. doi: 10.1034/j.1399-3054.2001.1120213.x 11454230

[B9] Liu W. (2020). Interfruit : deep learning network for classifying fruit images. bioRxiv. doi: 10.1101/2020.02.09.941039

[B10] Lu J. Lee W. Gan H. Hu X. (2018). Immature citrus fruit detection based on local binary pattern feature and hierarchical contour analysis. Biosyst. Engineering. 171, 78–90. doi: 10.1016/j.biosystemseng.2018.04.009

[B11] Lu J. Sang N. Hu Y. Fu H. (2014). Detecting citrus fruits with highlight on tree based on fusion of multi-map. J. Light-and Electronoptic. 125, 1903–1907. doi: 10.1016/j.ijleo.2013.04.135

[B12] Lu J. Yang R. Yu C. Lin J. Chen W. Wu H.. (2022). Citrus green fruit detection via improved feature network extraction. Front. Plant Sci. 13, 946154. doi: 10.3389/fpls.2022.946154 36578336 PMC9791251

[B13] McCool C. Perez T. Upcroft B. (2017). Mixtures of lightweight deep convolutional neural networks: applied to agricultural robotics. IEEE Robotics Automation Letters. 2, 1344–1351. doi: 10.1109/LRA.2017.2667039

[B14] Ning Z. Luo L. Ding X. Dong Z. Yang B. Cai J.. (2022). Recognition of sweet peppers and planning the robotic picking sequence in high-density orchards. Comput. Electron. Agriculture. 196, 106878. doi: 10.1016/j.compag.2022.106878

[B15] Ojala T. Pietikainen M. Maenpaa T. (2002). Multiresolution gray-scale and rotation invariant texture classification with local binary patterns. IEEE Trans. Pattern Anal. Mach. Intell. 24, 971–987. doi: 10.1109/TPAMI.2002.1017623

[B16] Pei Y. He C. Liu H. Shen G. Feng J. (2022). Compositional analysis of four kinds of citrus fruits with an NMR-based method for understanding nutritional value and rational utilization: from pericarp to juice. Molecules 27, 2579. doi: 10.3390/molecules27082579 35458777 PMC9031779

[B17] Peng H. Zou X. Xiong J. Chen Y. Guo A. Chen K. (2014). Recognition of mature citrus in natural scene under the occlusion condition. J. Inf. Comput. Science. 11, 1947–1958. doi: 10.12733/issn.1548-7741

[B18] Qiang L. Cai J. Bin L. Lie D. Zhang Y. (2014). Identification of fruit and branch in natural scenes for citrus harvesting robot using machine vision and support vector machine. Int. J. Agric. Biol. Engineering. 7, 115–121. doi: 10.3965/j.ijabe.20140702.014

[B19] Romero P. Lafuente M. (2020). Abscisic acid deficiency alters epicuticular wax metabolism and morphology that leads to in-creased cuticle permeability during sweet orange (Citrus sinensis) fruit ripening. Front. Plant Science. 11, 594184. doi: 10.3389/fpls.2020.594184 PMC7755607 33362823

[B20] Sharan L. Liu C. Rosenholtz R. Adelson E. (2013). Recognizing materials using perceptually inspired features. Int. J. Comput. Vision 103, 348–371. doi: 10.1007/s11263-013-0609-0 PMC3728085 23914070

[B21] Sun Y. Singh Z. Tokala V. Heather B. (2019). Harvest maturity stage and cold storage period influence lemon fruit quality. Scientia Horticulturae. 249, 322–328. doi: 10.1016/j.scienta.2019.01.056

[B22] Wang S. Xie W. Yan X. (2022). Effects of future climate change on citrus quality and yield in China. Sustainability 14, 9366. doi: 10.3390/su14159366

[B23] Wang Z. Xun Y. Wang Y. Yang Q. (2022). Review of smart robots for fruit and vegetable picking in agriculture. Int. J. Agric. Biol. Engineering. 15, 33–54. doi: 10.25165/j.ijabe.20221501.7232

[B24] Xiong J. Zheng Z. Liang J. Zhong Z. Liu B. Sun B. (2020). Citrus detection method in night environment based on improved YOLO v3 network. Trans. Chin. Soc. Agric. Mach. 51, 199–206. doi: 10.6041/j.issn.1000-1298.2020.04.023

[B25] Xu X. Ding Y. Lv Z. Li Z. Sun R. (2023). Optimized pointwise convolution operation by Ghost blocks. Electronic Res. Archive. 31, 3187–3199. doi: 10.3934/era.2023161

[B26] Xu L. Zhu S. Chen X. Wang Y. Kang Z. Huang P.. (2020). Citrus recognition in real scenarios based on machine vision. DYNA 95, 87–93. doi: 10.6036/DYNAII

[B27] Yang R. Hu Y. Yao Y. Gao M. Liu R. (2022). Fruit target detection based on BCo-YOLOv5 model. Mob. Inf. Syst. 2022. doi: 10.1155/2022/8457173

[B28] Yang C. Xiong L. Wang Z. Wang Y. Shi G. Kuremot T.. (2020). Integrated detection of citrus fruits and branches using a convolutional neural network. Comput. Electron. Agric. 174, 105469. doi: 10.1016/j.compag.2020.105469

[B29] Yi T. Zhang D. Luo L. Luo J. (2024). View planning for grape harvesting based on active vision strategy under occlusion. IEEE Robot. Autom. Lett. 9, 2535–2542. doi: 10.1109/LRA.2024.3357397

[B30] Zhao C. Lee W. He D. (2016). Immature green citrus detection based on colour feature and sum of absolute transformed difference (SATD) using colour images in the citrus grove. Comput. Electron. Agric. 124, 243–253. doi: 10.1016/j.compag.2016.04.009

