# Supplementary material for: Fusion of fruit image processing and deep learning: a study on identification of citrus ripeness based on R-LBP algorithm and YOLO-CIT model
Source: Front Plant Sci. 2024 Jun 5;15:1397816. doi: 10.3389/fpls.2024.1397816 (PMC11188418; doi:10.3389/fpls.2024.1397816)
Supplement: Supplementary file 1 [file Table_1.docx]

## Supplementary Tables

**Table 1.** Basic training set composition.

| **Image Type** | **Num** |
| --- | --- |
| Medium distance exposure citrus image | 318 |
| Medium range natural light citrus image | 361 |
| Medium distance backlight citrus image | 355 |
| Close range exposure citrus image | 264 |
| Close range natural light citrus image | 290 |
| Close range backlight citrus image | 266 |
| Background image | 197 |
| total | 2051 |
